# Supplementary material for: Design, Synthesis, and Biological Evaluation of N-Acylhydrazones and Their Activity Against Leishmania amazonensis Promastigotes
Source: Microorganisms. 2025 Jul 2;13(7):1563. doi: 10.3390/microorganisms13071563 (PMC12298860; doi:10.3390/microorganisms13071563)
Supplement: Supplementary file 1 [file microorganisms-13-01563-s001.zip › HPLC amostras-20241114T144635Z-001.zip!/HPLC amostras/HPLC composto 37 - PMM 44 - 18.pdf]

# Farmanguinhos - Fiocruz
# Serviço de Métodos Analíticos

PMM 44 - 18 - Rep 2.lcd
2018 - Sintese 1 - João Mafra - 2-5/2-204-4 - PMM 44 - 18 - Rep 2.lcd
25/06/2018, 06:46:14

Sample Name : PMM 44 - 18 - Rep 2
Sample ID : 118772
Sample Full Path : 2018 - Sintese 1 - João Mafra - 2-5/2-204-4 - PMM 44 - 18 - Rep 2.lcd
Data Filename : PMM 44 - 18 - Rep 2.lcd
Method Filename : Nitroimidazol.lcm
Batch File : 2018 - Sintese 1 - João Mafra - CLAE-10 - CLAE 10_2018 _0619.lcb
Vial # : 1-71 Sample Type : Unknown
Injection Volume : 10 uL
Date Acquired : 25/06/2018 06:46:43 Acquired by : Marcio Soares
Date Processed : 27/06/2018 09:16:51 Processed by : Marcio Soares
Data Comment : Método: Nitroimidazol

Preparo da Amostra: 1,35 mg foram adicionados 4 mL de metanol, agitada em vortex e levada ao ultrassom por 3 min. Posteriormente filtrada em membrana PVDF de 0,22µm e injetada.

Method

<<Header>>

<<Pump>>
Total Flow : 1,0000 mL/min
Pump A : LC-20ADXR

<<Mobile Phase Name>>
Pump A Mobile Phase A : Água
Pump A Mobile Phase C : Acetonitrila

<<LC Time Program>>

| Time | Module | Command | Value | C |
|---|---|---|---|---|
| 0,01 | Pumps | Solvent C Conc. | 20 | |
| 0,01 | Pumps | Total Pump A Flow | 1 | |
| 5,00 | Pumps | Solvent C Conc. | 20 | |
| 22,00 | Pumps | Solvent C Conc. | 90 | |
| 25,00 | Pumps | Solvent C Conc. | 90 | |
| 33,00 | Pumps | Solvent C Conc. | 20 | |
| 39,00 | Pumps | Solvent C Conc. | 20 | |
| 39,01 | Controller | Stop | | |

<<Data Acquisition>>
LC Stop Time : 39,01 min

<<Oven>>
Oven Temperature : 30 C

Instrumentation

<<Instrument>>

<<Pump A>>
Pump A Type : LC-20ADXR

<<System Controller>>
SCL Type : CBM-20A

<<Autosampler>>
Autosampler : SIL-30AC

<<Oven>>
Oven Type : CTO-20AC

<<Column>>
Column Name : Supelcosil LC- 8 - 5 µm
Column ID : CL 0173
Length : 150 mm
Internal Diameter : 4,6 mm
Max Number of Injections : 9999
Expiration Date : 28/04/2015
Max Temperature : 80,0 C
Installation Date : 28/04/2030

<<PDA>>
Detector Name : PDA
Detector Type : SPD-M20A

<<Degasser>>
Degasser : DGU-20A5R

# Farmanguinhos - Fiocruz
# Serviço de Métodos Analíticos

PMM 44 - 18 - Rep 2.lcd
2018 - Sintese 1 - João Mafra - 2-5/2-204-4 - PMM 44 - 18 - Rep 2.lcd
25/06/2018, 06:46:14

Chromatogram

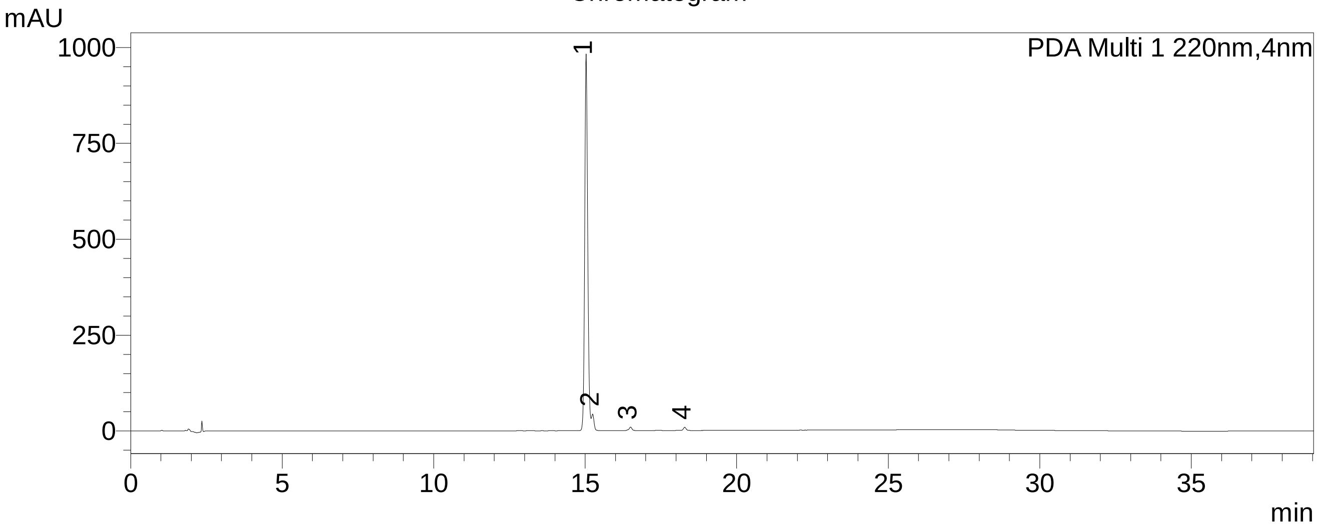


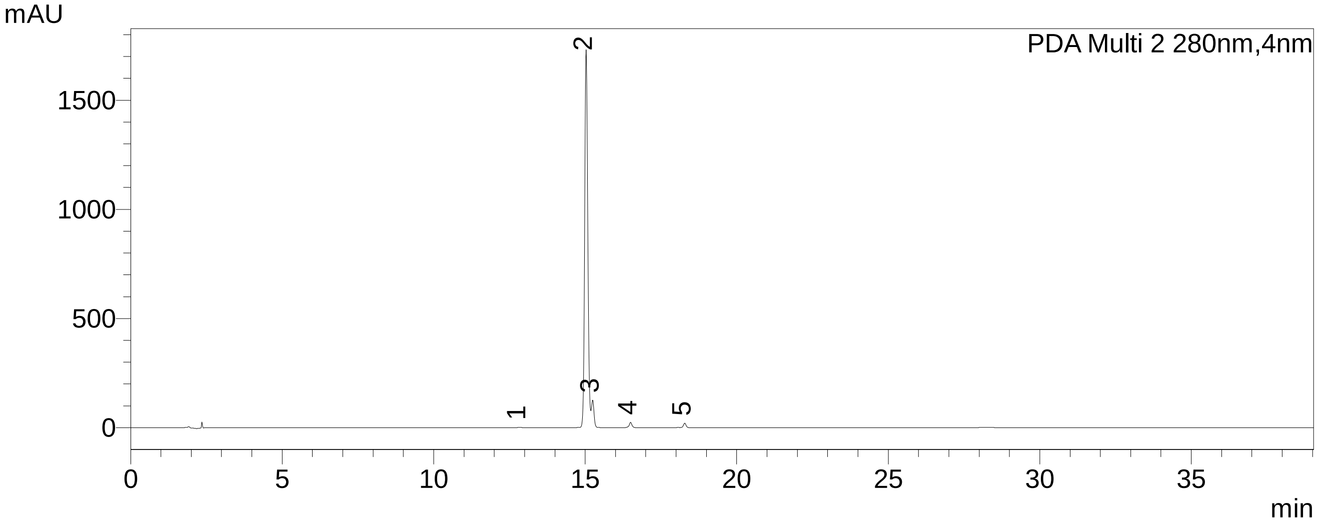


Peak Table

PDA Ch1 220nm

| Peak# | Ret. Time | Name | Area | Area% | Theoretical Plates/meter(USP) | Tailing Factor | Resolution(USP) | Capacity Factor(k') |
|---|---|---|---|---|---|---|---|---|
| 1 | 15,03 | | 6767856 | 94,8 | 611816 | 1,148 | -- | -- |
| 2 | 15,25 | | 255676 | 3,6 | 332275 | -- | 0,924 | 0,014 |
| 3 | 16,50 | | 64528 | 0,9 | 761562 | 0,900 | 5,350 | 0,098 |
| 4 | 18,28 | | 49971 | 0,7 | 1090818 | 1,034 | 9,480 | 0,216 |
| Total | | | 7138031 | 100,0 | | | | |

PDA Ch2 280nm

| Peak# | Ret. Time | Name | Area | Area% | Theoretical Plates/meter(USP) | Tailing Factor | Resolution(USP) | Capacity Factor(k') |
|---|---|---|---|---|---|---|---|---|
| 1 | 12,84 | | 13870 | 0,1 | 330294 | 0,995 | -- | -- |
| 2 | 15,03 | | 12474154 | 92,3 | 574048 | 1,173 | 10,072 | 0,171 |
| 3 | 15,25 | | 739076 | 5,5 | 497117 | -- | 1,021 | 0,188 |
| 4 | 16,50 | | 161681 | 1,2 | 831475 | 0,921 | 6,105 | 0,285 |
| 5 | 18,28 | | 124502 | 0,9 | 1096291 | 1,035 | 9,706 | 0,424 |
| Total | | | 13513283 | 100,0 | | | | |
